# Lung volume distribution in prolonged mechanical ventilation patients from assist control mode to spontaneous trial mode of automatic tubing compensation in electrical impedance tomography

**DOI:** 10.1186/2197-425X-3-S1-A666

**Published:** 2015-10-01

**Authors:** MY Chang, HT Chang

**Affiliations:** Far Eastern Memorial Hospital, Division of Pulmonary Medicine, Taipei City, Taiwan Province of China; Institute of Medical Engineering and Materials Science, National Yang-Ming University, Taipei, Taiwan Province of China; Far Eastern Memorial Hospital, Critical Care Medicine, New Taipei City, Taiwan Province of China

## Introduction

Electrical impedance tomography (EIT) is a non-invasive and portable lung imaging technique for dynamic evaluation of lung volume distribution. Previous studies had proved the EIT can provide information on regional distribution of ventilation and changes in end-expiratory lung volume (△EELV). The effect of EIT application in ventilation distribution of prolonged mechanical ventilation (PMV) patients underwent weaning program is unknown.

## Objectives

As an evidence-based strategy to predict successful weaning from assisted ventilation, automatic tube compensation (ATC) was developed to overcome the imposed work of breathing and airway resistance of endotracheal tube. The aim of the present study was to compare the spatial and temporal differences of ventilation distribution between control ventilation mode and 100% ATC in (PMV) patients.

## Methods

PMV **p**atients were ventilated under volume assist-control (AC) mode and subsequently under 100% ATC weaning. Spatial and temporal ventilation distributions were monitored with EIT. EIT data under AC mode 5 minutes before the switch to ATC and 50 minutes after switch were analyzed. We compared the data of end-expiratory lung impedance change (ΔEELI), ventilation distribution in regions of interest (ROIs), ratio of tidal variation, the global inhomogeneity (GI) index, the center of ventilation (CoV) index, regional ventilation delay (RVD) index of the lung regions in AC group and ATC group of the RCC population.

## Results

A total of 16 PMV patients ( MV>21 days) in respiratory care center (RCC)were includedTidal variations during ATC100% were significantly smaller than that during AC (*p* < 0.001). Regional ventilation distributions moved significantly towards dorsal regions during ATC100% ( EIT-based index center of ventilation, AC vs. ATC: 46.2 ± 5.8 vs. 51.7 ± 6.5, *p* < 0.001). Reginal ventilation delays were significantly reduced at ATC (50.2 ± 10.3 vs. 39.3 ± 6.9 in dorsal regions, *p < 0.001*).

## Conclusions

We found significant difference in ΔEELI, ROIs, RVDI, GI index, and CoV in 100%ATC trial as compared with AC mode. In conclusion, with EIT monitoring, we found ATC effectively stimulating the dorsal phrenic nerve to improve respiratory muscle weakness in PMV patients.

## Grant Acknowledgment

This work was financially supported by project Far Eastern Memorial Hospital (FEMH-2015-C-069). The authors would like to thank Zhanqi Zhao for his continuous, enthusiastic, and smart support for this article.
